# Improved mineralization of dental enamel by electrokinetic delivery of F^−^ and Ca^2+^ ions

**DOI:** 10.1038/s41598-022-26423-4

**Published:** 2023-01-10

**Authors:** NamBeng Tay, HiongYap Gan, Frederico Barbosa de Sousa, Lu Shen, Diego Figueiredo Nóbrega, Chenhui Peng, LaTonya Kilpatrick-Liverman, Wei Wang, Stacey Lavender, Shira Pilch, Jongyoon Han

**Affiliations:** 1 Engineering Cluster, Singapore Institute of Technology, 10 Dover Drive, Singapore, 138683 Singapore; 2 Department of Morphology and Graduate Program in Dentistry, Health Sciences Center, Federal University of Paraiba, Joao Pessoa, Cidade Universitaria, Paraíba, Brazil; 3 Cesmac University Center, Professional Masters Research in Health, Maceió, AL Brazil; 4 Department of Physics and Materials Science, University of Memphis, Memphis, TN USA; 5 Colgate-Palmolive Technology Center, Piscataway, NJ USA; 6 Department of Electric Engineering and Computer Science, Massachusetts Institute of Technology, Cambridge, MA USA; 7 Department of Biological Engineering, Massachusetts Institute of Technology, Cambridge, MA USA

**Keywords:** Microfluidics, Nanopores, Dental treatments

## Abstract

This in vitro study evaluated the effects of the infiltration of F^-^ and Ca^2+^ ions into human enamel by electrokinetic flow (EKF) on the enamel microhardness and F^-^ content. Sound human enamel ground sections of unerupted third molars were infiltrated with de-ionized water by EKF and with F^-^ ion by EKF respectively. All samples were submitted to two successive transverse acid-etch biopsies (etching times of 30 s and 20 min) to quantify F^-^ ion infiltrated deep into enamel. Remarkably, sound enamel showed a large increase in microhardness (MH) after infiltration of NaF (p < 0.00001) and CaCl_2_ (p = 0.013) by EKF. Additionally, NaF-EKF increased the remineralization in the lesion body of artificial enamel caries lesions compared to controls (p < 0.01). With the enamel biopsy technique, at both etching times, more F^-^ ions were found in the EKF-treated group than the control group (p << 0.05), and more fluoride was extracted from deeper biopsies in the NaF-EKF group. In conclusion, our results show that EKF treatment is superior in transporting Ca^2+^ and F^−^ ions into sound enamel when compared to molecular diffusion, enhancing both the mineralization of sound enamel and the remineralization of artificial enamel caries.

## Introduction

Our modern way of living has greatly reduced oral mechanical forces, which are used to perform natural tooth cleaning, and provided higher sugar consumption, responsible for cariogenic acid formation in oral biofilms. These have contributed to the surge of dental caries as the most prevalent chronic disease in human worldwide^1–4^. It has been shown that caries prevalence in adults is ten-fold higher than that experienced by children, even in developed countries^1,2^. In addition, enamel can be affected by the process of chemical dissolution by acids that are not produced by oral bacteria (dental erosion), which is also quite prevalent (4–83% in European adults)^5^. Together with the increasingly aging population, the maintenance of enamel integrity during a lifetime is a challenging and unsolved clinical problem in dentistry.

As the first layer of defense against dental caries, dental enamel is known as the most mineralized tissue of the human body, mostly composed of long and thin hydroxyapatite crystallites (70 nm × 30 nm in cross-section, extending from the enamel-dentine junction to the enamel surface). The narrow nanochannels separating those crystallites (2–3 nm in both prismatic and interprismatic regions, and 4–10 nm in the prism sheath; the latter is the main transport route) impose a serious challenge to the transport of materials. This is particularly challenging for negatively charged materials (e.g. fluoride ions) because their transport is hampered by the electric double layer that covers hydroxyapatite crystallites' surfaces and accounts for most of the inter-crystalline space in narrow nanochannels^6–8^. The dissolution of enamel due to caries and erosion could be hindered by transporting relevant ions to hydroxyapatite solubility (such as Ca^2+^ and F^−^) deep into normal (sound) enamel sublayers. Nevertheless, existing caries prevention methods are limited to a diffusion process that delivers ions very slowly, at unpredicted rates, and to the outermost enamel layer only^9–12^.

To overcome the difficulty of diffusion through the nanometer-sized pores^7^, a novel method of enhancing transport of materials into teeth is highly desirable. As an alternative, using electric fields to enhance the transport of materials into enamel has been investigated by different groups. Ivanoff and coworkers used dielectrophoresis to transport fluoride and carbamide peroxide into the enamel to the depth of 50–100 μm by applying an alternating current (AC) electric field^10,13^. Iontophoresis was used by Pitts and coworkers to deliver remineralizing agents into minor enamel surface lesions^14^. However, in the transport within micro- and nanoporous material such as teeth enamel or porous rock, it is widely known that electrokinetic flow (EK) is the dominant mechanism^15^. EK flow is the motion of liquid induced by an applied potential across a porous material, and this has been widely utilized in micro-/nanofluidic systems for decades^16^. This net fluid flow is created by the charged fluid layer near the surface (also known and the Debye layer), and the flow speed of EK flow is independent of the pore size but only depends on the surface charge density. Therefore, it is ideally suited for driving foreign materials into the narrow, nanometer size pores of teeth enamel. This mechanism is different from dielectrophoresis^10,13,17^ because it does not require AC electric field nor field gradient. EK flow-based infiltration is differentiated from iontophoresis^18^, because the EK flow delivers the entire fluid column into the pore (both positive and negative, as well as neutral components of the fluid), regardless of the charge state of the agent to be delivered. Therefore, the application of EK flow has shown a greater promise in enhancing material transport into the pores of normal enamel^19–21^. It was first demonstrated in Gan et al*.*^19^ that full replacement of the water content of a millimeter length of ground enamel sheet with a concentrated salt aqueous solution (Thoulet’s solution) could be done within 180 min, compared to a month needed to replace only 10% of water content by diffusion. However, whether the microhardness of enamel can be improved after such infiltration, which is the most critical function of the enamel layer in vivo, remain unelucidated.

Here, the aims of this in vitro study were to assess the effect (1) of applied electrical field strength using sound and artificially carious enamel, and (2) the association of low concentration F^-^ and Ca^2+^ ionic solutions, on the enamel mineralization (evaluated by micro-hardness, MH) by the treatment with solutions NaF and CaCl_2_. Using microhardness (MH) as the figure of merit (instead of whole teeth) is necessary because natural teeth enamel is inherently heterogeneous, both among different teeth samples as well as different locations of the same enamel sample. The hypotheses tested, by comparing the EKF and molecular diffusion methods, were that (1) the MH of enamel samples that were subjected and not subjected to an electrical field is similar, (2) EKF treatment with low-F^−^ and low-Ca^2+^ solutions would strengthen the hardness of enamel when compared to the baseline.

## Results

### Microhardness (MH) testing

Based on hardness testing data shown in Supplementary Information Note 5^22,23^, normal enamel MH values measured before and after infiltration of CaCl_2_ by EKF resulted in a medium correlation (Pearson R = 0.486; 95% CI = 0.70−0.20; power of 88.5%; p < 0.002). Infiltration of CaCl_2_ into normal enamel by EKF resulted in an increase in MH with a medium effect size as compared to the baseline (Hedge’s g of 0.652; 95% CI = 0.306−1.000; power of 80.0%; p = 0.013) (refer to Supplementary Information Note 5^22,23^ for statistical analysis of MH).

As for the infiltration of NaF, a stronger correlation (Pearson R = 0.53; 95% CI = 0.74−0.22; power of 90%; p < 0.002) was observed between MH values measured before and after the infiltration of NaF. Compared to baseline values (measured before NaF infiltration), normal enamel MH presented a large increase (Hedge’s g of 1.308; 95% CI = 0.916−1.70; power of 99.9%; p < 0.00001) after infiltration of NaF by EKF. The increase was observed even in the inner enamel layer (refer to Supplementary Information Note 5^22,23^ for statistical analysis of MH changes in relation to enamel depth).

### Bland and Altman’s plot

**Figure 1:**
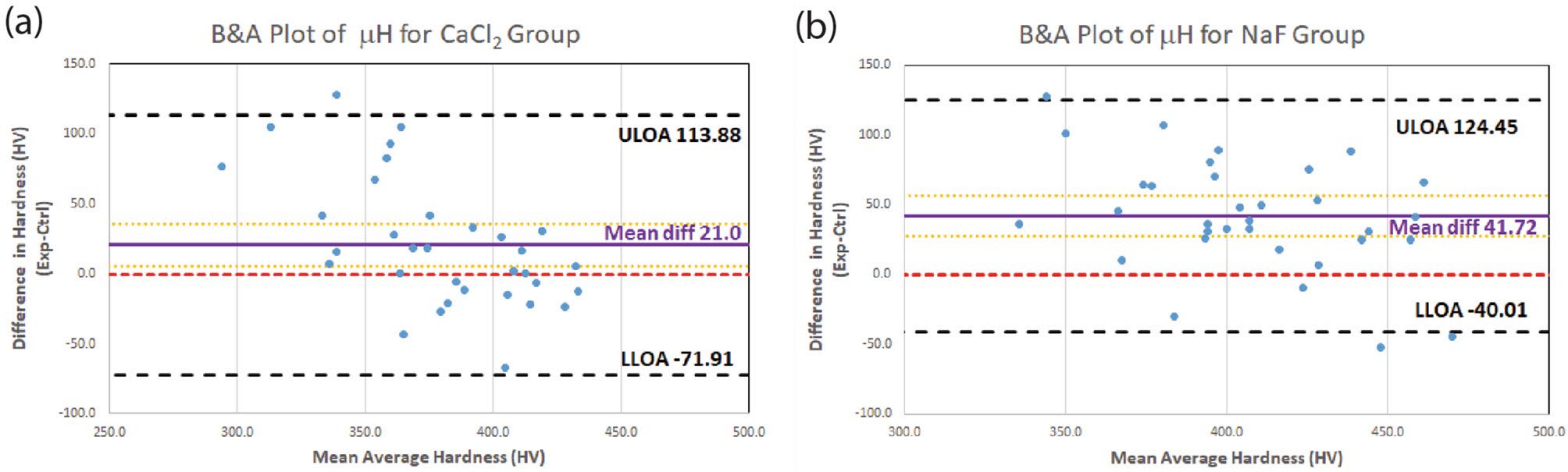
Agreement (Bland & Altman) plot of the difference in microhardness (in HV values) (EXP–CTRL) against their mean average (experimental & control) for (**a**) the CaCl_2_ group and (b) the NaF group. Short-dash red line represents equality; Solid purple line represents the Mean difference in hardness; Dotted orange lines represent 95% CI limit; Black dash lines represent the limits of agreement (LOA) with 95% CI. In (**a**) one outlier positive value was found, while in (**b**) two outlier negative values were found.

Compared Fig. 1a and b, both NaF and CaCl_2_ EKF-infiltrated groups with normal enamel had shown a positive mean difference in MH before and after EKF treatments. Particularly, the NaF group showed a higher positive mean difference than the CaCl_2_ group, which is aligned with the reported results^24^. (Refer to Supplementary Information Note 5^22,23^ for Bland and Altman analysis data).

### Remineralization (ΔMH%) of artificial enamel caries

EKF-driven infiltration of NaF into artificial enamel caries resulted in higher remineralization (ΔMH of 29% ± 12.4%) in the body of the lesion compared to the other groups (diffusion-driven infiltration of NaF, with ΔMH of − 4.33% ± 5.04%; and EKF-driven infiltration of DI water, with ΔMH of 4.12% ± 12.12%), with high effect sizes (Hedge´s g > 2.0; p < 0.01). Despite a slight increase of mean MH was observed for EKF-driven infiltration of DI water. The ANOVA analysis showed 95% confidence no difference between EKF-driven infiltration of DI water and diffusion-driven infiltration of NaF^20^ (see details in Supplementary Information Note 3).

### Fluoride ion measurement

All hypothesis testing cases showed a higher fluoride concentration in enamel after EFK treatment when compared to the control group (normal enamel immersed in de-ionized water), see Table 1. NaF-EKF presented a higher amount of F^-^ ion extracted from normal enamel using both acid biopsy times of 30 s (large effect size of 2.27, 95% CI of 1.277–3.262) and 20 min (large effect size of 1.68, 95% CI of 0.806–2.548). In addition, as the biopsy depth increased, more F^-^ ions from NaF-EKF treated samples than from the control group, with a large effect size of 1.535 (95% CI of 0.840–2.231). Additional experiments showed that even compared to NaF 10 mM solution (infiltration by passive diffusion, without EKF, upon application on the enamel surface by diffusion for 2 h), the NaF-EKF groups resulted in more F^-^ ions is extracted from normal enamel after etching time of 30 s (see Supplementary Information Note 4^25–28^). The effectiveness of EKF in delivering F^-^ in comparison to the diffusion method can also be found in Supplementary Information Note 4^25–27^.

**Table 1:** Hypothesis testing results of three different test cases.

Hypothesis testing case		No. of sample (n)	Mean (SD) μg F/cm^2^	Test method	P value	Hedge’s g	95% CI	Power
I	G1—DI Water	14	μ_1_ = 2.369 (0.414)	Two-sample T test	0.00000107 (reject H_0_)	2.27	1.277, 3.262	> 0.99
	G2—NaF by EKF	15	µ_2_ = 4.709 (1.375)					
II	G3—DI Water	16	μ_1_ = 3.171 (1.407)	Two-sample T test	0.0000798 (reject H_0_)	1.677	0.806, 2.548	> 0.99
	G4—NaF by EKF	15	µ_2_ = 5.743 (1.659)					

			Median (IQR) μg F/cm^2^					
III	ΔF_1_ = G3–G1	14	μ_1_ = 0.140 (0.545)	Mann–Whitney test	0.007762 (reject H_0_)	1.535	0.840, 2.231	> 0.84*
	ΔF_2_ = G4–G2	15	µ_2_ = 1.010 (0.885)					

Statistical analysis of F^−^ ion quantification (μg F/cm^2^) from normal enamel (I) using acid biopsy for 30 s; (II) using acid biopsy for 20 min; Statistical analysis of delta-F^−^ (ΔF^−^) ion quantification (μg F/cm^2^) (III) from normal enamel using acid biopsy. Case (I): NaF infiltration by EKF does not affect the amount of F ion incorporated into normal enamel quantification as compared with DI infiltration by EKF; Case (II): The depth of the enamel biopsy does not affect the amount of F ion extracted from EKF-treated normal enamel; Case (III): No effect from the diffusion treatment of NaF and the electrical field of EKF on enamel.

*1-tailed 5% significance level.

## Discussion

For the first time in the literature, we show improved microhardness (MH) of normal enamel deep into the enamel layer in response to the infiltration of Ca^2+^ and F^−^ ions from the original tooth surface to the enamel layer inward^29^. This has implications in the prevention of enamel dissolution resulting from both dental caries and erosion. Our results, control experiment (DI water by EKF), do not show any negative effect of EKF in normal enamel MH. We hypothesize that the organic coatings of hydroxyapatite crystallites, which play an important role in enamel MH^30^, were not removed by EKF. (Refer to Supplementary Information Note 6).

Measured MH values are consistent with values reported previously in the literature for normal enamel in human mature permanent teeth^31^. Furthermore, the corresponding EKF electrical current response was consistent with earlier reports^19^.

The present results indicate that EKF treatments significantly increased fluoride ion concentration in treated enamel, representing superior transportation of external molecules or ions when compared to the molecular diffusion method. Therefore, the null hypothesis was rejected (p << 0.05). In addition to increasing mineral content in normal enamel, increased remineralization in the body of the lesion of artificial enamel caries was achieved by EKF-driven infiltration of NaF (5 V/mm for 2 h) compared with diffusion-driven infiltration of NaF for 2 h. While a single application of NaF by diffusion is not representative of real-life conditions, where repetitive exposure to low doses of fluoride is the common practice, the effect on the lesion body (depth of 40–60 μm) is an indication of higher availability provided by EKF treatment of fluoride ions deep into the enamel caries lesion body after a single NaF application. In this context, it is important to note that a single application (5 min) of a high-dose fluoride solution (487 mM of amine fluoride) followed by saliva immersion for 21 days remineralized only the surface layer (depth of 25 μm) of artificial enamel caries in bovine enamel (3 times more permeable than the human enamel used here)^32^.

The improved MH values observed after infiltration of both solutions can be explained based on the solubility constant of hydroxyapatite and the ionic activity product of the solution that fills the pores between the hydroxyapatite crystallites' walls of the enamel nanochannels. We assume that the baseline condition in the enamel pores was that of equilibrium between the hydroxyapatite mineral [Ca_10_(PO_4_)_6_(OH)_2_] and the solution in the pores (i.e., ionic activity product equals the solubility constant). Considering that enamel hydroxyapatite has a solubility product of 10^–117.8^ M at room temperature^33^, approximately 4.7 × 10^–7^ M of Ca^2+^ would be dissolved in the solution filling the enamel pores. Regarding PO_4_, the concentration would be ≥ 2.7 × 10^–7^ M. In saliva, which is supersaturated with regard to hydroxyapatite, the mean calcium concentration is 1 mmol/L^34^. EKF-driven infiltration of Ca^2+^ into enamel pores increased Ca^2+^ concentration, which, in turn, increased the ionic activity product in the solution, making it supersaturated with regard to hydroxyapatite EKF. This resulted in mineral precipitation, increasing MH values. Most likely, the flow induction occurred mostly in larger pores (prism sheaths), which account for a small fraction of the total pore content, and the infiltrated ions later diffused into the larger pore fraction composed of smaller pores (inside both prismatic and interprismatic enamel), where previously found Ca^2+^and PO_4_ combined with recently infiltrated Ca^2+^and F^−^ ions, resulting in mineral precipitation. This is consistent with previous data showing that recently EKF-driven infiltration of F^−^ ions bound to fluoride fluorescent probes previously found in enamel pores (Peng et al.^20^). Here no attempts were undertaken to increase PO_4_ concentration into larger pores, and this could explain why the increase in post-EKF MH values had a medium effect size (Hedges’ g of 0.46).

The higher increase in post-EKF MH values observed in the F^−^ group can be explained by the lower solubility constant of fluorapatite [Ca_10_(PO_4_)_6_F_2_], which is 119.2 (100 times lower than that of HA)^33^. Compared to hydroxyapatite, fluorapatite requires lower concentrations of Ca^2+^ (~ 80% of that required to achieve saturation with regard to hydroxyapatite) and PO_4_ ions (~ 80% of that required to achieve saturation with regard to hydroxyapatite) in the surrounding solution to achieve saturation. F^−^ ions infiltrated (0.01 M, corresponding to 190 ppm) in the enamel pores were probably mixed with the Ca^2+^ and PO_4_ concentrations for saturation with regard to hydroxyapatite. This would have resulted in the super-saturation of the pore solution with regard to fluorapatite, resulting in fluorapatite precipitation. Thereby, no calcium fluoride deposits are expected to be formed in the enamel pores as a result of the EKF-driven fluoride infiltration because the solubility product constant of calcium fluoride is much higher (10^–10.45^ M)^35^ than that of hydroxyapatite. This means that the calcium concentration needed to achieve saturation with regard to calcium fluoride is much higher than that needed to achieve saturation with regard to hydroxyapatite in enamel pores^36^. Considering the CaF_2_ solubility constant (10.45)^35^, the assumed baseline calcium concentration in enamel pores (10^–7^ M), and the fluoride concentration in the test solution (0.01 M; 190 ppm), the calculated ionic activity product in the enamel pores after EKF was ~ 10^–11^ M, which is lower than the CaF_2_ solubility product, thus so being unsaturated. Thus, EKF-driven fluoride infiltration resulted in the precipitation of fluorapatite on the nanochannels walls and enriched the enamel pores with free fluoride ions. Such a modified sound enamel might be a slow-release source of fluoride ions able to protect the enamel from future carious and or erosive challenges. According to our assumptions described above, both Ca^2+^ and PO_4_ concentrations at baseline were higher than that needed for saturation with regard to fluorapatite, and this could explain why the increase in post-MH values had a large effect size (Hedges’ g of 1.31). It must be emphasized that our interpretation is based on the KsP values of HAP, FAP and CaF_2_, on the amount of Ca^2+^ and F^−^ infiltrated, and on changes in microhardness and enamel biopsy results. The true nature of the chemical composition of the formed minerals was not determined here, consisting in a limitation of this study, and further studies are required to elucidate this issue.

The explanation given above holds only if the EKF treatment resulted in the test solutions being mixed with the solution-filling enamel pores at baseline. That is, there was a partial replacement of the original enamel water content by the test solutions. Given the different pore sizes in normal enamel (2–3 nm in interprismatic enamel, and 4–10 nm in prisms’ sheaths)^37^, where the EKF rate is expected to be dependent to pore sizes^38^, probably mixture of test and original solutions occurred in the smaller pores. Regarding carious enamel, infiltrated fluoride ions are expected to be released slowly over time, which might provide a larger remineralization effect than that reported here. Mineral changes measured at longer post-EKF treatment times should be investigated and compared with remineralization resulting from repetitive ordinary low-dose fluoride application on the enamel surface, as the latter is the most used fluoride application in real life.

There is evidence indicating that the removal of organic matter increases enamel microhardness^30^. The displacement of the water content in the enamel nanochannels could, in theory, displace enamel organic content and, thus, contribute to the increase in post-EKF MH values. To eliminate this possibility, we tested changes in enamel MH after EKF-driven infiltration of DI water, which does not contribute to increasing ionic activity product either of hydroxyapatite or fluorapatite. Our results show a similarity between baseline and post-EKF treatment with DI water, suggesting that removal of organic matter was negligible if any, and probably did not contribute to the observed increase in enamel post-EKF MH.

Mean human enamel fluoride content is low and does not change with age, with major variations in enamel fluoride content being restricted only to the outermost surface layer (~ 20 microns^39^, where a fluoride-enriched zone can be found in response to fluoride incorporation from oral biofilm^40^. The continuous wear to which enamel is exposed throughout life progressively exposes the fluoride-poor inner enamel^40^, which is more susceptible to caries^41^ and affects oral health more intensively as one age. In this context, EKF-driven fluoride infiltration deep into the enamel layers might be a novel, highly beneficial measure to improve public oral health measures in similar way vaccines are applied to combat different diseases. This would have the advantage of not increasing fluoride levels in the blood, avoiding any side-effect related to systemic fluoride exposure.

It is worth noting that EKF can be applied clinically in Dentistry, similarly to the electronic root apex locator system that explores the relationship between applied voltage and measured electric current^42^, but with the difference that the anode would be on the tooth surface and the cathode on the oral mucosa surface (for example, on the lower mouth vestibule). Future studies are required to test this possibility.

In conclusion, EKF-driven infiltration of ions enhanced normal enamel microhardness, increased fluoride content deep into the normal enamel layer, and improved remineralization of artificial enamel caries. Considering the very low diffusion of negative ions in nanochannels with negatively charged walls, the protective effect of fluoride ions infiltrating deep into the enamel layer might last for a long period.

## Material and methods

All procedures involving human participants included in this study were in accordance with ethical standards. Unerupted human third molars (n = 16) from healthy patients (aged 18–30 years) were obtained from the Department of Morphology and Graduate Program in Dentistry, Health Sciences Center, Federal University of Paraiba, Joao Pessoa, Cidade Universitaria, Paraiba, Brazil during 2016–2017 after approved by the local institutional ethics committee for research on human beings (Lauro Wanderley University Hospital, Federal University of Paraiba, João Pessoa, Paraiba, Brazil), under the CAAE number 38199414.4.0000.5188). All volunteers were informed for the purposes of the research and signed an informed consent document and a tooth donation term sheet.

### Experimental design

**Figure 2:**
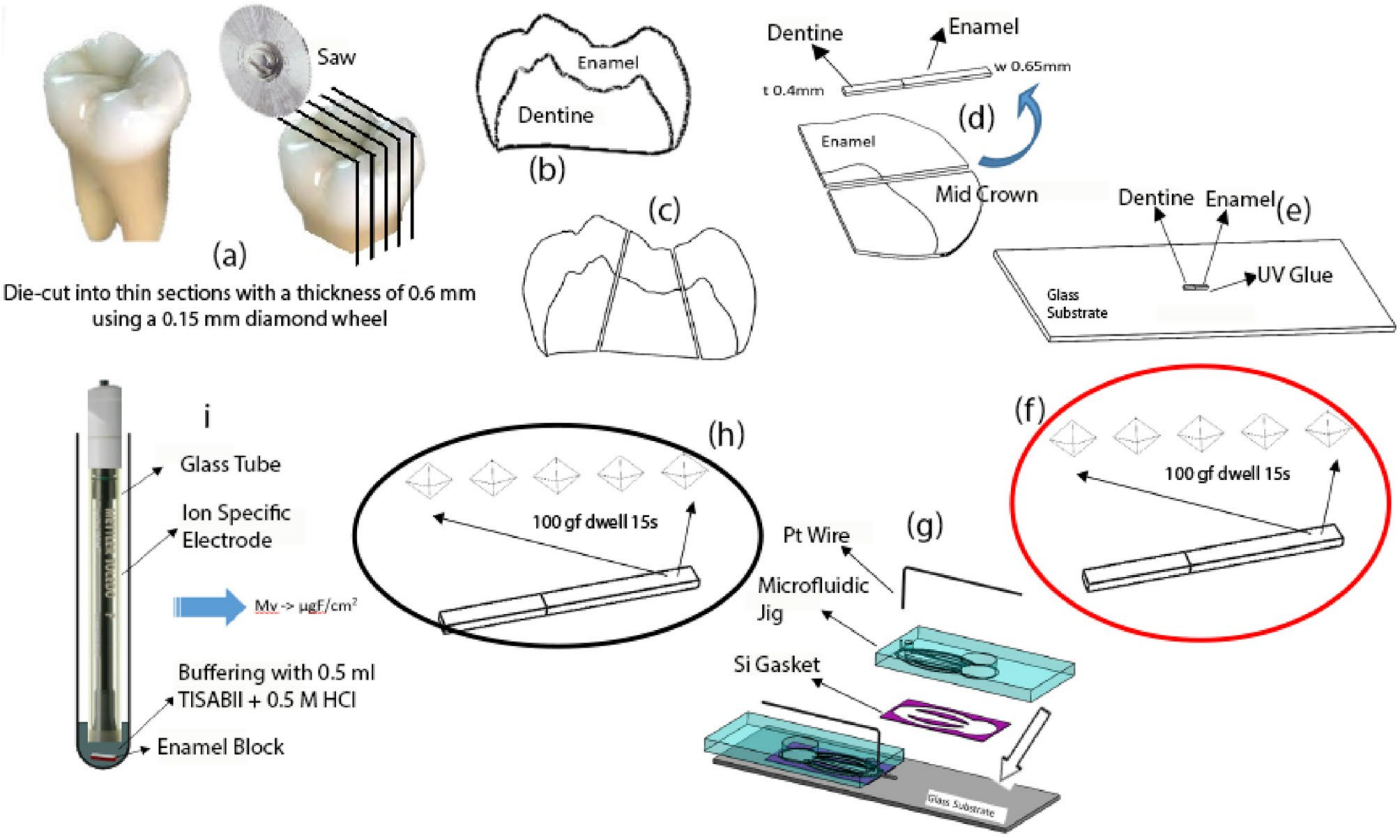
Preparation of specimen and experimental design. (**a**), (**b**) The tooth sample is die-cut into thin sections, which include the whole tooth crown. (**c**), (**d**) Each ground sheet is removed from part of its dentine and extracted from the buccal and lingual surfaces of the mid-crown region. (**e**), (**f**) Each enamel ground section is glued on a standard glass slide. The ground section sample is then measured for baseline MH at varying distances from the original enamel surface. (**g**) Subsequently, set it up in the microfluidic chip for specific EKF treatment. (**h**) Post-MH test is then performed on the treated sample section. (**i**) Lastly, the sample was submitted for Fluoride ion concentration measurement (in μg F/cm^2^).

The sample was composed of unerupted human third molars with completed roots with no signs of hypo-mineralization, staining, or morphological malformations. After extraction, debris and soft-tissue remnants were removed with periodontal curettes, and with gauze soaked with distilled water (pH 7), and then the samples were stored in an aqueous solution of sodium azide 0.02% (pH 7) until the experimental procedures were performed. Whole tooth samples, Fig. 2a, are then die-cut into thin sections with a thickness of 0.6 mm using a 0.15 mm diamond disc mounted in a sawing machine under constant water irrigation, and each section included the whole tooth crown as illustrated in Fig. 2b. Then, each section sample is ground to a thickness of 0.4 mm in a polishing machine using silicon carbide paper (2000 grit sizes). Subsequently, enamel ground section samples (0.65 mm in width) are extracted from the buccal and lingual surfaces of the mid-crown region of a ground section sample (Fig. 2c, d). Each ground enamel section then adheres on a standard 1 mm thick glass slide by using UV-curable glue (Optical Adhesive 68, Norland), see Fig. 2e.

Due to the considerable variations in enamel microhardness (MH) as a function of the location of the tooth crown^43^, baseline measurements of the MH are obtained before the EKF treatment. The enamel ground section samples (0.65 mm width × 0.4 mm thick) are first submitted to microhardness testing, Fig. 2f, and then mounted on a microfluidic chip, Fig. 2g (see the detailed setup in Supplementary Information Note 1), for EKF treatment using 5 V/mm for 180 min, similar to the procedures described previously^19^. Aqueous solutions of CaCl_2_ (10 mM) and NaF (10 mM), and deionized water, used close to neutral pH, were infiltrated into the enamel by EK field. Subsequently, the EKF-treated samples are submitted for final MH testing, Fig. 2h. Lastly, samples are retrieved from the glass slide for fluoride ion concentration measurement, see Fig. 2i. While for the molecular diffusion experiment, the specimen was set up on a microfluidic chip with the same test solutions filling up the inlet and outlet reservoirs with no electrical potential for 180 min.

### Microhardness (MH) testing

Before the Vickers hardness test, each of the ground enamel section samples was first hydrated in a deionized water bath for 24–36 h. MH testing was performed on each ground section sample before (CTRL) and after EKF treatment (EXP). MH was measured on the cut surface of samples (perpendicular to the original enamel surface). For normal enamel specimen, a sequence of 10 indentations was created 100 μm apart at different distances (100, 200, 300, 400, 500, 600, 700, 800, 900, and 1000 μm) from the outer enamel surface using a DURAMIN-40 M1 hardness tester (Struers ApS, Ballerup, Denmark). After EKF treatment, using either solutions of CaCl_2_ (10 mM), NaF (10 mM), or deionized (DI) water (pH of 6.5–7.0), for 2 h, a new row of 10 indentations was made, 100 μm distant from the baseline measurements and at the same distances from the enamel surface. Similarly for specimens with artificial caries lesions, but the indentation locations were adjusted to accommodate the depth of the lesion layer (~ 80 to 100 µm). 3 groups of 3 indentations were created within the lesion layer, parallel to the enamel surface. Pre-treatment indentations were located ~ 20 µm from the enamel surface, while the post-treatment indentations were located ~ 50 µm apart from pre-treatment indentations (see the details in Supplementary Information Note 3). Artificial enamel caries lesions were prepared as described previously^19^, and details of preparing the artificial enamel caries lesion can be found in Supplementary Information Note 2.

The effect of EKF treatment on the remineralization (quantified as the percentage of MH values difference, ∆MH%) of artificial enamel caries (body of the lesion) was also statistically tested with control experiments (see Supplementary Information Note 3^25^).

### Microhardness (MH) statistical analysis of normal enamel

As mineral volume varies considerably along the enamel layer^22^, resulting in high statistical variability, a single MH value is not representative of the whole enamel layer, so various points of measurement are required for evaluating MH changes. Cluster analysis was used (hierarchical complete linkage method^44^ to test the dependency of different histological points along the enamel layer (from the surface to the enamel-dentine junction). Based on measured data before EKF, five cluster reference values were calculated: minimum, percentile 25%, percentile 50%, percentile 75%, and maximum.

Each enamel sample, comprised of its MH values, was submitted to cluster analysis at a time. Data composed by all MH values of each enamel sample, and the five reference cluster values were submitted for cluster analysis using hierarchical clustering, and sub-type complete linkage. Up to five MH values could be selected from each enamel sample. After cluster analysis, selected data comprised the final sample size submitted to statistical analysis of the hypotheses of correlation and difference, and the test of agreement (Bland & Altman analysis).

### Bland–Altman (B-A) analysis

In B-A analysis, a scatter plot is constructed in which the difference between the paired measurements is plotted on the y-axis and the average of the measures of the two methods on the x-axis^24,45,46^. The mean difference in values obtained with the two methods is called the bias and is represented by a central horizontal line on the plot. The standard deviation (SD) of differences between paired measurements is then used to construct horizontal lines above and below the central horizontal line to represent 95% limits of agreement (LOA) (mean bias ± 1.96 SD) and is called upper and lower LOA.

### Fluoride ion concentration analysis by enamel biopsy measurement

Two groups (control and experimental) of enamel ground sections were used in the fluoride concentration analysis. The control group was enamel samples immersed in a microcentrifuge tube (1.5 ml, Eppendorf) with deionized (DI) water for at least 120 h, while the experimental group was infiltrated with 10 mM NaF by EKF treatment. Two consecutive layers of enamel were acid extracted by immersing each ground section sample in 0.5 ml of an aqueous solution of 0.5 M hydrochloric acid (HCl, Acid fuming 37%, ACS reagent, Sigma-Aldrich, Singapore) for the 30 s and 20 min, respectively, under agitation. After the extraction time, an equal volume of TISAB II with CDTA (Ionic strength adjustment (ISA) solution, Mettler Toledo, Singapore), pH 5.0, modified with 0.5 M NaOH (M = 40 g/mol, 98.5–100.5%, VMR International, Singapore), was immediately added to each solution containing the dissolved enamel layer. Fluoride measurement is then performed using an ion-specific electrode (pH/Ion Meter S220, Mettler Toledo). The amounts of fluoride found were expressed as micrograms of fluoride per square centimeter of dissolved enamel section area (μg F/cm^2^). More details can be found in Supplementary Information Note 4^25–28^.

Comparisons between the two groups were performed with a 5% significance level (2-tailed), and effect size (Hedges’ g), its 95% confidence interval, and power were calculated^25^. For non-normally distributed data, Hedges’ g was calculated by using medians and 75% of interquartile ranges instead of means and standard deviations^28^. Data were analyzed for the presence of extreme outliers. That is, those values lower than the 25th quartile minus 3 × interquartile range (IQR) or greater than the 75th quartile plus 3 × IQR^47^. Extreme outliers are expected to be found once per 450,000 observations in normally distributed data^47^. Only upper-extreme outliers were found.

## Supplementary Information

Supplementary Information.

## Data Availability

All data generated or analyzed during this study are included in the supplementary information file.
