# Supplementary material for: Improved mineralization of dental enamel by electrokinetic delivery of F− and Ca2+ ions
Source: Sci Rep. 2023 Jan 10;13:516. doi: 10.1038/s41598-022-26423-4 (PMC9832158; doi:10.1038/s41598-022-26423-4)
Supplement: Supplementary file 1 — Supplementary Information. [file 41598_2022_26423_MOESM1_ESM.docx]

**SUPPLEMENTAL INFORMATION**

**Improved mineralization of dental enamel by electrokinetic delivery of F^-^ and Ca^2+^ ions**

NamBeng Tay^1^, HiongYap Gan^1,†^*, Frederico Barbosa de Sousa^2,†^, Lu Shen ^1^, Diego Figueiredo Nóbrega^3^, Chenhui Peng^4^, LaTonya Kilpatrick-Liverman^5^, Wei Wang^5^, Stacey Lavender^5^, Shira Pilch^5^ and Jongyoon Han^6,7^*

^1^ ^1^ Engineering Cluster, Singapore Institute of Technology, 10 Dover Drive, Singapore 138683

^2^ Department of Morphology and Graduate Program in Dentistry, Health Sciences Center, Federal University of Paraiba, Joao Pessoa, Cidade Universitaria, Paraiba, Brazil

^3^ Cesmac University Center, Professional Masters Research in Health, Maceió, AL, Brazil

^4^ Department of Physics and Materials Science, University of Memphis, Memphis, TN, USA

^5^ Colgate-Palmolive Technology Center, Piscataway, NJ, USA

^6^ Department of Electric Engineering and Computer Science, Massachusetts Institute of Technology, Cambridge, MA, USA

^7^ Department of Biological Engineering, Massachusetts Institute of Technology, Cambridge, MA, USA

^†^ These authors contributed equally to this work.

*Corresponding author: hiongyap.gan@singaporetech.edu.sg and [jyhan@mit.edu](mailto:jyhan@mit.edu)

**Keywords**

dental caries; nanotechnology; fluorides; tooth remineralization; electrokinetic flow; microhardness

SUPPLEMENTAL INFORMATION

**Note 1:** Electrokinetic Flow Experiment Setup

Each of the strip specimens, which consists of a full normal enamel portion at mid-crown area and part of the dentine portion, was examined under a stereomicroscope. Specimen with any apparent stains, cracks, or chips was discarded. Then, dentine was removed, and the ready strip specimen (strip specimen on glass slide) was then soaked in de-ionized water for at least 24h^[[1]](#footnote-1)^ before pre-EKF hardness measurement. Subsequently, the specimen was assembled on a microfluidic flow jig for the EK-infiltration experiment.

The microfluidic flow jig, see Figure 1a & 1c, consists of a microfluidic chip, a thin hydrophobic elastomer (a.k.a. gasket), and 4× binder clips. Four reservoirs were built into the microfluidic chip (Figure 1b) where inner two small working reservoirs were placed at both ends of the strip specimen and the other two supplementary reservoirs were connected to the working reservoirs via internal connecting channels. The purpose of supplementary reservoirs was to minimize the water level drop in the working reservoir during infiltration operation for 180 minutes. Two platinum (Pt) wires were permanently secured at the working reservoirs respectively to minimize potential disturbances (such as position, location, etc.) of measurement during EK infiltration.

EKF infiltration experiments on a fully assembled test device (Figure 1c) was carried out with a Keithley digital sourcemeter 2450 (Keithley Inc., USA) connected via triax cable (Keithley Inc. USA) to the two Pt electrodes, which served as anode and cathode respectively located at both ends of strip specimen. Keithley KickStart control software was used with the source meter to control the required applied voltage (5 V/mm in flow length) for electrokinetic pumping, and to record the corresponding system current and resistance during EK experiment at the acquisition of 1 data/minutes.

tooth surface

mucosa surface

cathode

anode


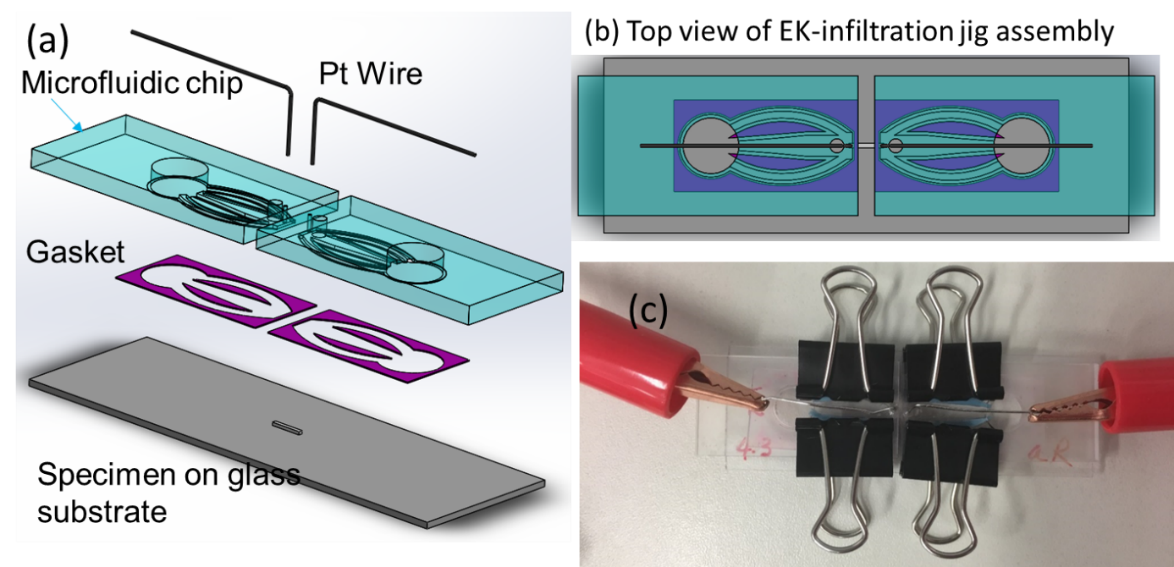


Figure 1. EK-treatment microfluidic device. (a) Exploded view of microfluidic jig assembly; (b) Top view of microfluidic jig; (c) Top view of fully assembled test device connected with Keithley triax cables.

**Note 2:** Artificial Enamel Caries Lesion Preparation

**2.1 Preparation of demineralization solution**

Demineralizing solution (final pH of 5.0) was prepared by adding 0.122 g of KH_2_PO_4_ (M^[[2]](#footnote-2)^ = 136.08 g/mol, 99.5-100.5%, VWR International, Singapore), 0.36 g of CaCl_2_.2H_2_O (M = 147.02 g/mol, ≥99.0%, Sigma-Aldrich, Singapore), 2.9 mL of acetic acid (M = 60.05 g/mol, the density of 1.05, ≥99.7%, Sigma-Aldrich, Singapore) to 800 mL of deionized water (DI).

**2.2 Artificial enamel caries induction**

A layer of acid-resistant nail varnish was applied on the entire enamel ground sheet (cut surfaces only) except the enamel surface. Artificial caries lesion formation was induced using a demineralization solution for 72h^[[3]](#footnote-3)^. Artificial carious enamel depth was 80 μm, as determined by digital microradiography using the digital X-ray of a microcomputed tomographic scanner, see Figure 2. (Skyscan model 1172, Bruker, Belgium; operation at 60 kV, with a pixel size of 0.9 μm).

Figure 2. Micro-radiographic images of the artificial enamel caries lesion of a tooth ground sheet specimen, showing subsurface demineralization (lesion body; radiolucent area) underneath a relatively unaffected surface layer (radiopaque area; arrow in B). Approximately 80 - 100 µm artificial carious enamel depth from the tooth surface.


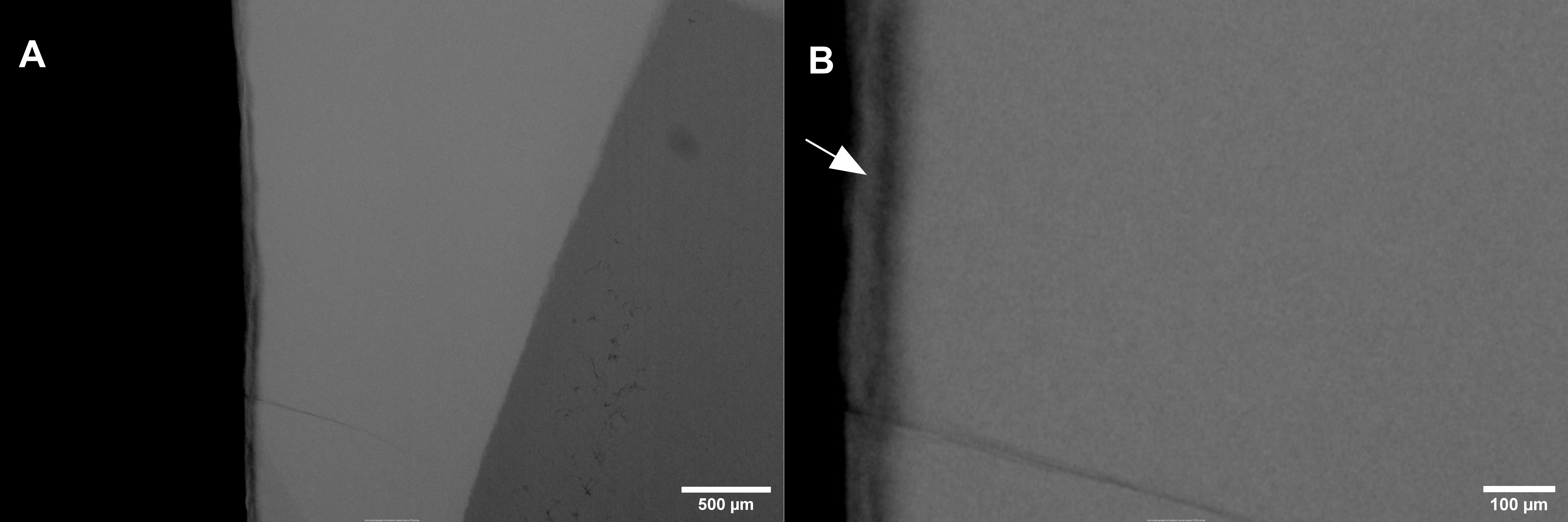


**Note 3:** Microhardness (MH^[[4]](#footnote-4)^) of Artificial Enamel Caries Lesions

**3.1 Microhardness measurement**

The following experimental groups were included: artificial enamel caries treated with NaF (10 mM^[[5]](#footnote-5)^, analytical standard (for ion-selective electrodes), Sigma-Aldrich, Singapore) via EKF (5 V/mm for 2h; NaF-EFK; n = 8); artificial enamel caries treated with NaF (10 mM) via diffusion (2h, without EKF; NaF-DIF), and artificial enamel caries treated with DI water via EKF (2h, DI-EKF; n = 7).

Transverse microhardness (Vickers) measurements were performed before and after treatment. In order to avoid the breakdown of the fragile carious enamel surfaces after successive mechanical indentations, indentations were performed only within the body of the lesion layer, that is ~ 80 to 100 µm. For each specimen, three sites were selected alongside the width of the specimen block (~0.65 mm), labeled as the top section, middle section, and bottom section. Each section is 250 µm apart, see Figure 3. At each site, three microhardness measurements were obtained, and a mean of nine measurements was used per specimen. Pre-treatment indentations were located ~20 μm from the enamel surface, while post-treatment indentations were located ~50 μm apart from pre-treatment indentations. The outcome was the ratio in percentage (ΔMH%)^[[6]](#footnote-6)^ of difference between pre-treatment MH (MH_0_) and post-treatment MH (MH_1_) values to MH_0_.


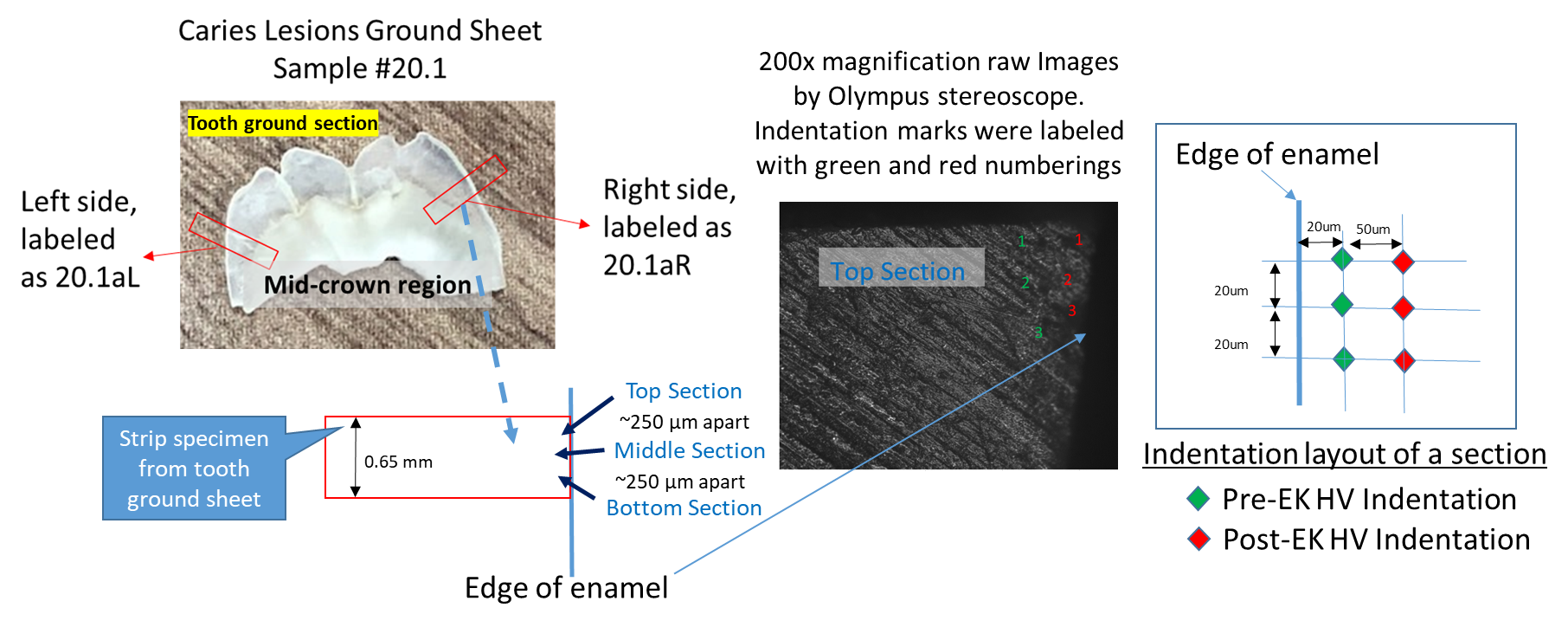


Figure 3. Micro-hardness indentation planning for test specimen (strip) within the body of the lesion layer. To provide a better view across the lesion of the strip specimen, three indentation sites were selected alongside the enamel surface. The three sites are named as top section, middle section, and bottom section respectively.

**3.2 Statistical analysis**

The null hypothesis was the treatment of artificial enamel caries (i.e. NaF by EKF, de-ionized water (DI) by EKF, and NaF by diffusion) has no effect on the remineralization (ΔMH%) of artificial enamel caries.

Statistical analysis was performed after testing for normality and homogeneity of variances as stated previously in this study. One-way ANOVA followed by pair-wise comparisons were performed using 5% significance level (1-tailed for ANOVA and 2-tailed for T test). The p-value, effect size (Hedges’ g), its 95% confidence interval, and power were calculated ^1^.

**3.3 Results**

The mean ± SD values (ΔMH%) were 29.15% ±12.40% (NaF-EKF), -4.33% ±5.04% (NaF-DIF), and 4.21% ±12.12% (DI-EKF). One-Way ANOVA results show the statistical difference with a large effect size (eta squared = 0.966), low p-value (4.05x10^-5^), and high power (>99.9%), thus rejecting the null hypothesis. The results pair-wise comparison is shown in Table 3.3.1. Compared with both NaF-DIF and DI-EKF groups, the EKF treatment with NaF 10 mM shows increased remineralization, with large effect sizes and high power (rejecting the null hypotheses).

Table 3.3.1. Results of statistical analysis of pair-wise comparisons on the remineralization (ΔMH%) of artificial enamel caries.

| ***Groups: NaF-EKF vs NaF-DIF*** | | | |
| --- | --- | --- | --- |
| p-value | Hedge´s g | 95% CI | Power |
| 5.4x10^-6^ | 3.544 | 1.825; 5.264 | >0.99 |
| ***Groups: NaF-EKF vs DI-EKF*** | | | |
| p-value | Hedge´s g | 95% CI | Power |
| 0.00172 | 2.035 | 0.659; 3.411 | >0.95 |
| ***Groups: NaF-DIF vs DI-EKF*** | | | |
| p-value | Hedge´s g | 95% CI | Power |
| 0.091 | 0.945 | -0.233; 2.124 | 0.37 |

**Note 4:** Fluoride Ion Concentration Analysis by Enamel Biopsy Measurement

Two groups (control and experimental) of enamel ground sections were used in fluoride concentration analysis. Control group was enamel samples immersed in microcentrifuge tube (1.5 mL, Eppendorf) with deionized (DI) water for at least 120h, while the experimental group was infiltrated with 10 mM NaF by EKF treatment. Two consecutive layers of enamel were acid extracted by immersing each ground section sample in 0.5 ml of an aqueous solution of 0.5M hydrochloric acid (HCl, Acid fuming 37%, ACS reagent, Sigma-Aldrich, Singapore) for 30s and 20min, respectively, under agitation. After the extraction time, an equal volume of TISAB II with CDTA (Ionic strength adjustment (ISA) solution, Mettler Toledo, Singapore), pH 5.0, modified with 0.5M NaOH (M = 40g/mol, 98.5-100.5%, VMR International, Singapore), was immediately added to each solution containing the dissolved enamel layer. Fluoride measurement is then performed using an ion-specific electrode (pH/Ion Meter S220, Mettler Toledo). The amounts of fluoride found were expressed as micrograms of fluoride per square centimeter of dissolved enamel section area (μg F/cm^2^).

All groups were tested regarding normality by calculating kurtosis and skewness, and those with a variation of ±2 from the optimal value (-2 to 2 in skewness; 1 to 5 in kurtosis) were considered as normally distributed ^2^. Test of homogeneity of variances was performed by calculating the ratio of the highest by the lowest group variances, and variances were considered homogeneous when such a ratio was ≤ 3 ^3^. Two-sample T-test was applied for data presenting normal distribution, otherwise the Mann-Whitney test was applied.

Comparisons between two groups were performed with a 5% significance level (2-tailed), and effect size (Hedges’ g), its 95% confidence interval, and power were calculated ^1^. For non-normally distributed data, Hedges’ g was calculated by using medians and 75% of interquartile ranges instead of means and standard deviations ^4^. Data were analyzed for the presence of extreme outliers. That is, those values lower than 25^th^ quartile minus 3× interquartile range (IQR) or greater than 75^th^ quartile plus 3× IQR ^5^. Extreme outliers are expected to be found once per 450,000 observations in normally distributed data ^5^. Only upper extreme outliers were found.

**4.1) Preparation of Calibration Solution for Fluoride Ion Selective Electrode**

Prior to each measurement, the apparatus (Mettler Toledo pH/Ion Meter S220) with ion-selective electrode (ISE) – F electrode was be calibrated with laboratory prepared calibration solutions. The calibration stock solution (20g/L of NaOH) was prepared by adding 2g of NaOH (M = 40g/mol, 98.5-100.5%, VMR International, Singapore) in 100 mL TISAB II with CDTA (Ionic strength adjustment (ISA) solution, Mettler Toledo, Singapore). Magnetic slow stirring was employed to ensure fully dissolved of NaOH. A series of dilution solutions (0.125 ppm, 0.25 ppm, 0.5 ppm, 1.0 ppm, 4.0 ppm, 16.0 ppm, 32.0 ppm) were prepared for obtaining F^-^ ion concentration calibration curve.

**4.2) Procedure of Enamel Biopsy Measurement**

Except for the enamel surface of interest (perpendicular to the original enamel surface), all enamel block surfaces were covered with an acid-resistant nail varnish. The exposed area was measured with digital caliper (precision of 0.01 mm). After the ISE F electrode was calibrated, the enamel biopsy was then performed by acid extraction with 0.5M HCl and TISABII at controlled exposure (etching) times. Two etching times, 30s and 20min, providing shallow and deep biopsies were tested, as described below.

**4.3) Hypotheses and Experiments**

The following hypotheses and corresponding experiments were performed:

1. Hypothesis 1 (normal enamel, biopsy with etching time of 30s): NaF infiltration by EKF does not affect the amount of F ion incorporated into normal enamel quantification as compared with DI infiltration by EKF. This was tested with an experiment including two treatment groups (DI water infiltration by EKF, n = 15; and NaF 10 mM infiltration by EKF, n = 16), using the same EKF treatment parameters described before. The post-treatment enamel etching time for collecting material for F ion quantification was 30s.
2. Hypothesis 2 (normal enamel, difference between biopsy with two etching times of 30s and 20min). As NaF infiltration by EKF is expected to have the highest impact on F ion incorporation in the bulk of the enamel block (where infiltration of NaF by diffusion is hindered), we tested the null hypothesis that depth of the enamel biopsy does not affect the amount of F ion extracted from EKF-treated normal enamel. The experiment included the same groups described in the previous paragraph (a), but now a new etching time of 20min was used and was compared with the 30s etching time.
3. Hypothesis 3 (normal enamel, NaF-EKF x NaF-DIF x DI-EKF): in order to test the effect of NaF (10 mM) treatment by diffusion, new samples were prepared (as described before) and allocated into the following groups (n = 3/group): NaF 10mM by EKF (5 V/mm) for 2 h; NaF-EKF), NaF 10 mM by applied by diffusion for 2 h, and DI applied by EKF (5 V/mm for 2 h). The enamel etching time 30s for all groups.

**4.4) Statistical Analysis**

All groups were tested regarding normality and homogeneity of variances as described above. T test was applied for data presenting nomal distribution, otherwise the Mann-Whitney test was applied. Comparisons between pairs of groups were performed with a 5% significance level (2-tailed), and effect size (Hedge’s G), its 95% confidence interval, and power were calculated ^1^. For non-normally distributed data, Hedge’s G was calculated by using medians and 75% of interquartile ranges instaead of means and standard deviations ^4^. Data were analyzed regarding the presence of extreme outliers, that is, those values lower than 25^th^ quartile minus 3× interquartile range (IQR) or greater than 75^th^ quartile plus 3 × IQR ^5^. Extreme outliers are expected to be found once per 450,000 observations in normally distributed data ^5^. Only upper extereme outliers were found.

**4.5) Results**

Results of F ion quantification in normal enamel are shown in Tables 4.5.1 (etching time of 30s; hypothesis 1), 4.5.2 (etching time 20 min), 4.5.3 (difference between two etching times of 30s and 20 min; hypothesis 2), and 4.5.4 (NaF-EKF x NaF-DIF x DI-EKF; hypothesis 3). The biopsy depths varied both within and between groups, with depths after 30s shallower than those after 20 min. The biopsy depths ranged from 20 μm to 65 μm. The more extreme depth values were found in the DI group.

Table 4.5.1. Raw data of F ion quantification (μg F/cm^2^) with 30s etching time.

| Control (DI-EKF) | Experiment (NaF-EKF) |
| --- | --- |
| 2.75 | 3.3 |
| 2 | 8.21 |
| 2.23 | 3.51 |
| 2.1 | 3.92 |
| 2.44 | 5.21 |
| 2.32 | 4.2 |
| 2.72 | 5.15 |
| 1.37 | 6.35 |
| 2.51 | 4.71 |
| 2.43 | 4.47 |
| 2.1 | 5.73 |
| **5.96** | 4.24 |
| 2.78 | 3.63 |
| 2.36 | 5.28 |
| **7.88** | 2.72 |
| 3.05 |  |
| **5.00** |  |

Extreme upper outliers: numbers in bold.

Table 4.5.2. Raw data of F ion quantification (μg F/cm^2^) with 20 mins etching time.

| Control (DI-EKF) | Experiment (NaF-EKF) |
| --- | --- |
| 2.82 | 5.27 |
| 2.17 | 10.09 |
| 2.28 | 4.36 |
| 1.93 | 4.57 |
| 2.28 | 5.51 |
| 2.39 | 4.36 |
| 2.83 | 6.59 |
| 1.43 | 7.94 |
| 3.18 | 5.32 |
| 2.92 | 4.98 |
| 2.68 | 7.42 |
| 6.92 | 5.25 |
| 3.89 | 4.69 |
| 3.93 | 5.96 |
| **10.17** | 3.84 |
| 3.44 |  |
| 5.65 |  |

Extreme upper outliers: numbers in bold.

Table 4.5.3. Raw data of ΔF (μg F/cm^2^) from two etching times, which is 30s in Table 4.5.1. and 20 min in Table 4.5.2.

| ΔControl (DI-EKF) | ΔExperiment (NaF-EKF) |
| --- | --- |
| 0.07 | 1.97 |
| 0.17 | 1.88 |
| 0.05 | 0.85 |
| -0.17 | 0.65 |
| -0.16 | 0.3 |
| 0.07 | 0.16 |
| 0.11 | 1.44 |
| 0.06 | 1.59 |
| 0.67 | 0.61 |
| 0.49 | 0.51 |
| 0.58 | 1.69 |
| 1.11 | 1.01 |
| 1.57 | 1.06 |
| 0.39 | 0.68 |
|  | 1.12 |

Regarding hypothesis 3, the descriptive data are shown in Table 4.5.4, One-Way ANOVA results and pair-wise comparisons are shown in Tables 4.5.5 and 4.5.6, respectively.

Table 4.5.4. Raw data of F ion concentration (μg F/g Enamel) using enamel biopsy measurement with 30s etching time.

| **Runs** | **NaF-EKF** | **DI-EKF** | **NaF-DIF** |
| --- | --- | --- | --- |
| **1** | 722.99 | 384.09 | 371.08 |
| **2** | 680.09 | 318.24 | 343.49 |
| **3** | 745.52 | 272.52 | 394.10 |
| ***Mean*** | *716.20* | *324.95* | *369.56* |
| ***SD*** | *33.243* | *56.088* | *25.341* |

Table 4.5.5. Results of One-way Anova for normal enamel groups.

| **p Value** | **eta squared (effect size)** | **Power** |
| --- | --- | --- |
| 4.04803x10^-5^ | 0.965 | > 99.99% |

The null hypothesis was rejected, with a large effect size and high statistical power.

Table 4.5.6. Results of statistical analysis of pair-wise comparisons on F ion concentration (μg F/g enamel; ppm) using acid biopsy.

| T test *1: NaF-EKF vs DI-EKF* | | | | | |
| --- | --- | --- | --- | --- | --- |
| P value | Hedge’s g | | 95% CI | | Power |
| 0.0019 | 8.486 | | 16.704/0.268 | | 0.997 |
| T test *2: NaF-EKF vs NaF-DIF* | | | | | |
| P value | | Hedge’s g | 95% CI | Power | |
| 0.000731 | | 11.728 | 22.811/0.645 | 0.9992 | |
| T test *3: DI-EKF vs NaF-DIF* | | | | | |
| P value | | Hedge’s g | 95% CI | Power | |
| 0.3361 | | 1.025 | 4.762/-2.712 | 0.0465 | |

**Note 5**: Microhardness (MH) Statistical Analysis of Normal Enamel

As mineral volume varies considerably along the enamel layer ^6^, resulting in high statistical variability, a single MH value is not representative of the whole enamel layer, so that various points of measurement are required for evaluating MH changes. We used cluster analysis (hierarchical complete linkage method ^7^ to test the dependency of different histological points along the enamel layer (from the surface to the enamel-dentine junction). Based on measured data before EKF, five cluster reference values were calculated: minimum, percentile 25%, percentile 50%, percentile 75%, and maximum. Each value represented a cluster. Each enamel sample, comprised by its MH values, was submitted to cluster analysis at a time. Data composed by all MH values of each enamel sample, and the five reference cluster values were submitted for cluster analysis using hierarchical clustering, sub-type complete linkage. Up to five MH values could be selected from each enamel sample. After cluster analysis, selected data comprised the final sample size submitted to statistical analysis of the hypotheses of correlation and difference, and the test of agreement (Bland & Altman analysis).

**5.1) Cluster Analysis for MH Measurement**

The procedures of cluster analysis were summarized in the following:

1. From MH values measured before EKF, five cluster reference values were calculated: minimum, percentile 25%, percentile 50%, percentile 75%, and maximum. Each value represented a cluster.
2. Each enamel sample, comprised by its MH values, was submitted to cluster analysis at a time. Data composed by all MH values of each enamel sample and the five reference cluster values were submitted for cluster analysis using hierarchical clustering, sub-type complete linkage. Up to 5 MH values could be selected from each enamel sample.
3. After cluster analysis, selected data comprised the final sample size submitted to statistical analysis of the hypotheses of correlation and difference, and the test of agreement (Bland & Altman plots).

Table 5.1.1. CaCl_2_ MH Data after cluster analysis.

| **Before** | **After** | **Before** | **After** |
| --- | --- | --- | --- |
| 429.4 | 434.71 | 260.81 | 365.62 |
| 389.91 | 416.32 | 439.08 | 426.93 |
| 359.31 | 377.75 | 402.8 | 419.43 |
| 274.62 | 402.89 | 312.51 | 353.93 |
| 438.49 | 371.12 | 392.56 | 372.03 |
| 394.77 | 382.99 | 406.92 | 409.24 |
| 363.05 | 363.55 | 375.41 | 408.17 |
| 347.53 | 375.28 | 313.13 | 406.08 |
| 255.47 | 332.64 | 425.41 | 403.31 |
| 439.4 | 416.33 | 364.96 | 383.72 |
| 388.13 | 382.86 | 330.48 | 346.71 |
| 311.44 | 416.63 | 419.46 | 413.53 |
| 412.97 | 398.28 | 392.52 | 366.03 |
| 403.86 | 434.11 | 317.14 | 400.04 |
| 386.45 | 343.23 | 384.02 | 374.61 |
| 332.35 | 340.08 | 425.64 | 409.24 |
| 412.14 | 412.75 | 396.19 | 408.17 |
| 354.5 | 396.17 | 352.27 | 360.36 |
| 320.12 | 387.13 | 381.27 | 394.27 |
|  |  |  |  |
|  |  |  |  |

Table 5.1.2 NaF MH Data after cluster analysis

| **Before** | **After** | **Before** | **After** |
| --- | --- | --- | --- |
| 428.4 | 459.4 | 394.6 | 482.4 |
| 473.8 | 421.7 | 429.4 | 454.4 |
| 343.6 | 389.1 | 375.9 | 412.1 |
| 385.9 | 435.4 | 352.6 | 441.8 |
| 361.1 | 431.1 | 317.6 | 353.1 |
| 280.3 | 407.4 | 428.0 | 493.7 |
| 354.5 | 434.7 | 380.4 | 406.1 |
| 407.2 | 425.1 | 342.1 | 405.9 |
| 380.0 | 428.2 | 428.6 | 418.7 |
| 492.0 | 447.8 | 326.5 | 433.7 |
| 425.1 | 432.0 | 387.8 | 463.0 |
| 387.8 | 426.0 | 444.7 | 469.1 |
| 390.8 | 423.4 | 378.5 | 409.2 |
| 362.4 | 372.3 | 399.0 | 368.5 |
| 299.3 | 400.3 | 401.6 | 454.4 |
| 438.0 | 478.9 | 345.1 | 407.9 |
|  |  | 383.7 | 416.1 |

**5.2) Statistical Analysis for MH Measurement ^1^**

Based on data showed below, normal enamel microhardness values measured before and after infiltration of CaCl_2_ by EKF resulted in a medium correlation (Pearson R = 0.49; 95% CI= 0.70 – 0.20; power of 88.5%; p < 0.002). Infiltration of CaCl_2_ into normal enamel by EKF resulted in an increase in microhardness (with a medium effect size: Hedge’s g of 0.652; 95% CI = 0.306 – 1.000; power of 80.0%; p = 0.013). For the infiltration of NaF, a stronger correlation (Pearson R = 0.53; 95% CI= 0.74 – 0.22; power of 90%; p < 0.002) between microhardness values measured before and after infiltration of NaF into normal enamel was found. Compared to baseline values (measured before NaF infiltration), normal enamel microhardness presented a large increase (Hedge’s g of 1.308; 95% CI = 0.916 – 1.70; power of 99.9%; p < 0.00001) after infiltration of NaF by EKF.

***5.2.1) CaCl_2_ Group***

*Table 5.2.1. Pearson Correlation (sample size = 38) - (5% 2-tailed significance level)*

| p-value | R coefficient | CI upper limit | CI Lower limit | Power |
| --- | --- | --- | --- | --- |
| 0.001961 | 0.486371 | 0.69759293 | 0.197370855 | 0.885373 |

*Table 5.2.2. Paired-difference (sample size = 38) – Paired T test (5% 2-tailed significance level)*

| p-value | Hedge g | CI Upper | CI Lower | Power |
| --- | --- | --- | --- | --- |
| 0.012799048 | 0.6528 | 1.0000 | 0.3055 | 0.8007 |

***5.2.2) NaF Group***

*Table 5.2.1 Pearson Correlation (sample size = 33) - (5% 2-tailed significance level)*

| p-value | R coefficient | CI upper limit | CI Lower limit | Power |
| --- | --- | --- | --- | --- |
| 0.001621736 | 0.527131 | 0.737059 | 0.224432 | 0.899014935 |

*Table 5.2.2. Paired-difference (sample size = 3) – Paired T test (5% 2-tailed significance level)*

| p-value | Hedge G | CI lim sup | CI lim inf | Power |
| --- | --- | --- | --- | --- |
| 0.00000256 | 1.3079 | 1.6997 | 0.9161 | 0.9994 |

**Note 6:** Analysis of Normal Enamel Microhardness (MH) Changes in terms of Depth (perpendicular to tooth surface) after F^-^ Ion Infiltration by EKF

Note: considering a mean enamel layer thickness (from the enamel surface to the enamel-dentin junction) of 1 mm. Enamel layer was divided into outer (100-300 µm from the enamel surface), middle (400-600 μm from the enamel surface), and inner enamel (700-900 µm from the enamel surface). Sample size was range from *n* = 28 to 33. **No cluster analysis was performed**.

**6.1) Outer Enamel (100-300 µm from the Enamel Surface)**

The tested hypothesis was that F^-^ ion infiltration by EKF affects enamel microhardness. Data were submitted to normality test. A paired T test, with 5% two-tailed significance level was applied. Results are shown in Tables 6.1.1 and 6.1.2.

Table 6.1.1. Descriptive statistics of outer enamel MH

|  | Before EKF | After EKF |
| --- | --- | --- |
| *mean* | 407.8542424 | 443.5351515 |
| *SD* | 34.58909538 | 29.45418212 |
| *n* | 33 | 33 |

Table 6.1.2. Results of Paired T test for outer enamel MH.

| **p-value** | **Hedge’s G** | **Upper 95% CI** | **Lower 95% CI** | **Power** |
| --- | --- | --- | --- | --- |
| 3.69087E-06 | 1.453 | 2.016 | 0.889 | >0.99 |

In conclusion: null hypothesis was rejected with a large effect size (1.453) and high power (>99.99%).

**6.2) Middle Enamel (400-600 µm from the Enamel Surface)**

The tested hypothesis was that F^-^ ion infiltration by EKF affects enamel microhardness. Data were submitted to normality test. A paired T test, with 5% two-tailed significance level was applied. Results are shown in Tables 6.2.1 and 6.2.2.

Table 6.2.1. Descriptive statistics of outer enamel MH.

|  | Before EKF | After EKF |
| --- | --- | --- |
| *Mean* | 386.2636364 | 416.6951515 |
| *SD* | 33.92580884 | 28.03522005 |
| *n* | 33 | 33 |

Table 6.2.2. Results of Paired T test for outer enamel MH.

| **p-value** | **Hedge’s G** | **Upper 95% CI** | **Lower 95% CI** | **Power** |
| --- | --- | --- | --- | --- |
| 0.000460491 | 1.042271124 | 1.576692823 | 0.507849426 | 0.99979877 |

In conclusion, the null hypothesis was rejected with a large effect size (1.042) and high power (99.98%).

**6.3) Inner Enamel (700-900 µm from the Enamel Surface)**

The tested hypothesis was that F^-^ ion infiltration by EKF affects enamel microhardness. Data were submitted to normality test. A paired T test, with 5% two-tailed significance level was applied. Results are shown in Tables 6.3.1 and 6.3.2.

Table 6.3.1. Descriptive statistics of outer enamel MH.

|  | Before EKF | After EKF |
| --- | --- | --- |
| *mean* | 367,1975 | 394,4203571 |
| *SD* | 33,76852552 | 34,36108269 |
| *n* | 28 | 28 |

Table 6.3.2. Results of Paired T test for outer enamel microhardness.

| **p-value** | **Hedge’s G** | **Upper 95% CI** | **Lower 95% CI** | **Power** |
| --- | --- | --- | --- | --- |
| 0,000717846 | 1,029567737 | 1,613142907 | 0,445992567 | 0,998934357 |

In conclusion, the null hypothesis was rejected with a large effect size (1.029) and high power (99.89%).

**References**

1 Cohen, J. *Statistical Power Analysis for the Behavioral Sciences*. (Routledge Academic, 1988).

2 Field, A. *Discovering Statistics Using SPSS*. 3rd edn, (Publisher: SAGE Publications Ltd;, 2009).

3 Dean, A. M. & Voss, D. *Design and Analysis of Experiments*. (Springer-Verlag New Yorkv, 1999).

4 Grissom, R. J. & Kim, J. J. *Effect Sizes for Research: Univariate and Multivariate Applications*. (Taylor & Francis, 2014).

5 Boslaugh, S. *Statistics in a Nutshell*. (O'Reilly Media, Incorporated, 2012).

6 Setally Azevedo Macena, M. *et al.* A comparative study on component volumes from outer to inner dental enamel in relation to enamel tufts. *Archives of Oral Biology* **59**, 568-577, doi:<https://doi.org/10.1016/j.archoralbio.2014.03.001> (2014).

7 Brian S., E., Sabine, L., Morven, L. & Daniel, S. *Cluster Analysis*. 5th edn, (John Wiley & Sons, Ltd 2011).

1. h represents hour(s) [↑](#footnote-ref-1)
2. M represents molar mass. [↑](#footnote-ref-2)
3. h represents hour(s) [↑](#footnote-ref-3)
4. MH represents microhardness. [↑](#footnote-ref-4)
5. mM represents millimolar concentration. [↑](#footnote-ref-5)
6. ΔMH% represents the ratio (in percentage) of difference between pre-treatment MH (MH_0_) and post-treatment MH (MH_1_) values to MH_0_ [↑](#footnote-ref-6)
